# Integrative Omics Uncovers Low Tumorous Magnesium Content as A Driver Factor of Colorectal Cancer

**DOI:** 10.1093/gpbjnl/qzae053

**Published:** 2024-07-25

**Authors:** Rou Zhang, Meng Hu, Yu Liu, Wanmeng Li, Zhiqiang Xu, Siyu He, Ying Lu, Yanqiu Gong, Xiuxuan Wang, Shan Hai, Shuangqing Li, Shiqian Qi, Yuan Li, Yang Shu, Dan Du, Huiyuan Zhang, Heng Xu, Zongguang Zhou, Peng Lei, Hai-Ning Chen, Lunzhi Dai

**Affiliations:** National Clinical Research Center for Geriatrics and General Practice Ward/International Medical Center Ward, General Practice Medical Center, State Key Laboratory of Biotherapy, West China Hospital, Sichuan University, Chengdu 610041, China; National Clinical Research Center for Geriatrics and General Practice Ward/International Medical Center Ward, General Practice Medical Center, State Key Laboratory of Biotherapy, West China Hospital, Sichuan University, Chengdu 610041, China; National Clinical Research Center for Geriatrics and General Practice Ward/International Medical Center Ward, General Practice Medical Center, State Key Laboratory of Biotherapy, West China Hospital, Sichuan University, Chengdu 610041, China; National Clinical Research Center for Geriatrics and General Practice Ward/International Medical Center Ward, General Practice Medical Center, State Key Laboratory of Biotherapy, West China Hospital, Sichuan University, Chengdu 610041, China; National Clinical Research Center for Geriatrics and General Practice Ward/International Medical Center Ward, General Practice Medical Center, State Key Laboratory of Biotherapy, West China Hospital, Sichuan University, Chengdu 610041, China; National Clinical Research Center for Geriatrics and General Practice Ward/International Medical Center Ward, General Practice Medical Center, State Key Laboratory of Biotherapy, West China Hospital, Sichuan University, Chengdu 610041, China; National Clinical Research Center for Geriatrics and General Practice Ward/International Medical Center Ward, General Practice Medical Center, State Key Laboratory of Biotherapy, West China Hospital, Sichuan University, Chengdu 610041, China; National Clinical Research Center for Geriatrics and General Practice Ward/International Medical Center Ward, General Practice Medical Center, State Key Laboratory of Biotherapy, West China Hospital, Sichuan University, Chengdu 610041, China; National Clinical Research Center for Geriatrics and General Practice Ward/International Medical Center Ward, General Practice Medical Center, State Key Laboratory of Biotherapy, West China Hospital, Sichuan University, Chengdu 610041, China; National Clinical Research Center for Geriatrics and General Practice Ward/International Medical Center Ward, General Practice Medical Center, State Key Laboratory of Biotherapy, West China Hospital, Sichuan University, Chengdu 610041, China; National Clinical Research Center for Geriatrics and General Practice Ward/International Medical Center Ward, General Practice Medical Center, State Key Laboratory of Biotherapy, West China Hospital, Sichuan University, Chengdu 610041, China; National Clinical Research Center for Geriatrics and General Practice Ward/International Medical Center Ward, General Practice Medical Center, State Key Laboratory of Biotherapy, West China Hospital, Sichuan University, Chengdu 610041, China; Institute of Digestive Surgery, West China Hospital, Sichuan University, Chengdu 610041, China; Colorectal Cancer Center, Department of General Surgery, West China Hospital, Sichuan University, Chengdu 610041, China; Advanced Mass Spectrometry Center, Research Core Facility, Frontiers Science Center for Disease-related Molecular Network, West China Hospital, Sichuan University, Chengdu 610041, China; National Clinical Research Center for Geriatrics and General Practice Ward/International Medical Center Ward, General Practice Medical Center, State Key Laboratory of Biotherapy, West China Hospital, Sichuan University, Chengdu 610041, China; National Clinical Research Center for Geriatrics and General Practice Ward/International Medical Center Ward, General Practice Medical Center, State Key Laboratory of Biotherapy, West China Hospital, Sichuan University, Chengdu 610041, China; Institute of Digestive Surgery, West China Hospital, Sichuan University, Chengdu 610041, China; Colorectal Cancer Center, Department of General Surgery, West China Hospital, Sichuan University, Chengdu 610041, China; National Clinical Research Center for Geriatrics and General Practice Ward/International Medical Center Ward, General Practice Medical Center, State Key Laboratory of Biotherapy, West China Hospital, Sichuan University, Chengdu 610041, China; Colorectal Cancer Center, Department of General Surgery, West China Hospital, Sichuan University, Chengdu 610041, China; National Clinical Research Center for Geriatrics and General Practice Ward/International Medical Center Ward, General Practice Medical Center, State Key Laboratory of Biotherapy, West China Hospital, Sichuan University, Chengdu 610041, China

**Keywords:** Magnesium deficiency, Colorectal cancer, Genome instability, Tumor metastasis, Phosphorylation

## Abstract

Magnesium (Mg) deficiency is associated with increased risk and malignancy in colorectal cancer (CRC), yet the underlying mechanisms remain elusive. Here, we used genomic, proteomic, and phosphoproteomic data to elucidate the impact of Mg deficiency on CRC. Genomic analysis identified 160 genes with higher mutation frequencies in Low-Mg tumors, including key driver genes such as *KMT2C* and *ERBB3*. Unexpectedly, initiation driver genes of CRC, such as *TP53* and *APC*, displayed higher mutation frequencies in High-Mg tumors. Additionally, proteomic and phosphoproteomic data indicated that low Mg content in tumors may activate epithelial–mesenchymal transition (EMT) by modulating inflammation or remodeling the phosphoproteome of cancer cells. Notably, we observed a negative correlation between the phosphorylation of DBN1 at S142 (DBN1^S142p^) and Mg content. A mutation in S142 to D (DBN1^S142D^) mimicking DBN1^S142p^ up-regulated MMP2 and enhanced cell migration, while treatment with MgCl_2_ reduced DBN1^S142p^, thereby reversing this phenotype. Mechanistically, Mg^2+^ attenuated the DBN1–ACTN4 interaction by decreasing DBN1^S142p^, which in turn enhanced the binding of ACTN4 to F-actin and promoted F-actin polymerization, ultimately reducing MMP2 expression. These findings shed new light on the crucial role of Mg deficiency in CRC progression and suggest that Mg supplementation may be a promising preventive and therapeutic strategy for CRC.

## Introduction

Metal ions are crucial for both physiological and pathological processes within living organisms [1,2]. Magnesium (Mg), a predominant intracellular divalent cation, is crucial for maintaining cellular homeostasis and participates in nearly all cellular processes [3,4]. The intracellular Mg content is up to 10–30 mM; however, the concentration of free Mg^2+^ in cells is only 0.5–1.2 mM [5]. Mg^2+^ can bind to ATP, ribosomes, or nucleotides as a cofactor, and serve as an activator of numerous enzymes involved in glycolysis, phosphorylation events, DNA repair, DNA stabilization, and protein synthesis [6–8]. Many proteins, such as MRS2, TRPM6/7, MAGT1, SCL41A1, and CNNMs, are well-established Mg^2+^ transporters [9]. Dysregulation of these transporters may lead to Mg^2+^ imbalance and associated diseases.

Mg is crucial for controlling cancer initiation and progression [5,10]. Disruptions in Mg homeostasis contribute to cancer progression by promoting the proliferation, angiogenesis, and invasion of cancer cells into surrounding tissues [11–13]. Additionally, changes in Mg levels can impair immune function, compromising the body’s ability to detect and eliminate cancerous cells [14,15]. Furthermore, Mg dysregulation affects chemoresistance, decreasing the susceptibility of cancer cells to chemotherapy [16,17]. Colorectal cancer (CRC) ranks as the third most common cause of cancer-related death globally. A meta-analysis of 29 studies demonstrated that increased Mg intake is linked to a reduced risk of CRC [18–21]. Previous investigations have shown that the anti-inflammatory properties of Mg may help reduce inflammation in the colon [22,23], which is a risk factor for CRC [24]. Moreover, Mg functions as an antioxidant, protecting colon cells against oxidative stress and preventing DNA damage [25–27]. In CRC treatment, Mg can enhance the effectiveness of chemotherapy by improving drug uptake by cancer cells and protecting healthy cells from damage [28].

Despite some progress, many questions about the role of Mg in CRC remain unanswered. First, there is a lack of systematic investigations on how Mg affects tumor progression at the molecular level. Second, it is unclear whether Mg has differential effects on left- and right-sided CRC at the molecular level [29]. Third, tumor metastasis is a leading cause of mortality in CRC patients. Mg deficiency not only reduces intracellular ATP levels [30] but also affects kinase and phosphatase activities [31,32]. Dysregulated protein phosphorylation is common in CRC and is linked to unfavorable outcomes [33–36]. However, the direct link between Mg deficiency and tumor metastasis through affecting protein phosphorylation still lacks direct evidence.

With the rapid development of omics technologies, the functional interpretation of metal ions during aging using omics data has been achieved [37]. In this study, we utilized an integrative genomic, proteomic, and phosphoproteomic approach to determine the role of Mg in CRC, and found that low Mg content in tumors affects both genomic stability and metastasis. Genomic analysis revealed that a low Mg content in tumors increased the frequency of gene mutations associated with tumor progression rather than initiation. Proteomic and phosphoproteomic analyses indicated that low Mg content in tumors could activate epithelial–mesenchymal transition (EMT) by activating the complement pathway and inducing inflammation or by directly remodeling protein phosphorylation in cancer cells. Furthermore, we demonstrated that Mg^2+^ weakened the interaction between DBN1 and ACTN4 by reducing the phosphorylation of DBN1 at S142 (DBN1^S142p^), which enhanced the interaction between ACTN4 and F-actin and promoted F-actin polymerization, ultimately leading to the down-regulation of MMP2 and reduced cancer cell migration.

## Results

### Low Mg content in tumors predicts an unfavorable prognosis in CRC patients

**Figure 1:**
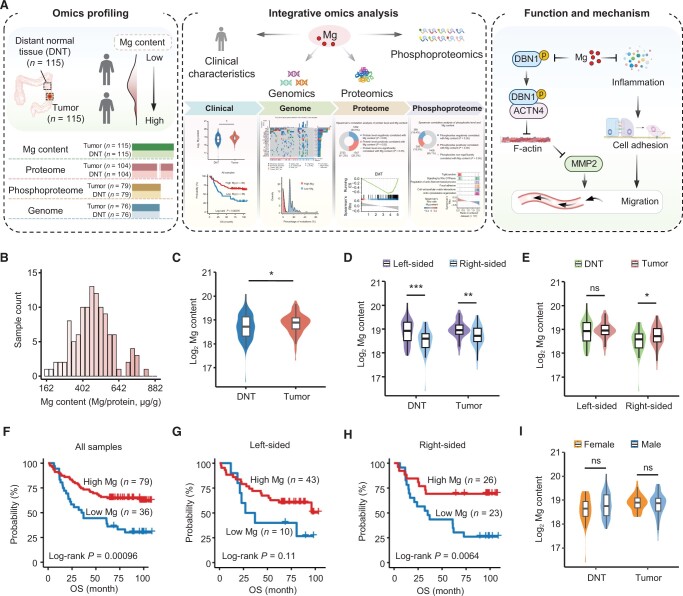
Low Mg content in tumors predicts an unfavorable prognosis in CRC patients **A**. Concept and workflow of this study. **B**. Intratumoral Mg content of CRCs. **C**. Comparison of Mg content between the tumors and paired DNTs of 115 CRCs. Wilcoxon rank-sum test (*, *P* < 0.05). **D**. Comparison of Mg content between left- and right-sided tumors or between left- and right-sided DNTs. Wilcoxon rank-sum test (***, *P* < 0.001; **, *P* < 0.01). **E**. Comparison of Mg content between tumors and paired DNTs located on the left or right side. Wilcoxon rank-sum test (*, *P* < 0.05; ns, no significance). **F**. Survival analysis of 115 CRC patients with different Mg concentrations in tumors. Log-rank test. **G**. Survival analysis of 53 left-sided CRC patients with different Mg concentrations in tumors. Log-rank test. **H**. Survival analysis of 49 right-sided CRC patients with different Mg concentrations in tumors. Log-rank test. **I**. Comparison of Mg content between females and males in tumors or DNTs. Wilcoxon rank-sum test (ns, no significance). Mg, magnesium; DNT, distant normal tissue; CRC, colorectal cancer; OS, overall survival.

To determine the clinical significance of low Mg content in tumors and its impact on tumor progression, omics data from 230 paired tissue samples collected from 115 treatment-naive CRC patients were used (**Figure 1**A; Table S1). Proteomic and phosphoproteomic analyses employed a tandem mass tag (TMT)-based quantitative approach (Figure S1A). Correlation analysis of quality control (QC) samples, internal standard (IS) samples for proteomics and phosphoproteomics, and replicate samples for proteomics demonstrated the stability of the instrument, as well as the reliability and reproducibility of the mass spectrometry (MS) data (Figure S1B–H). A total of 9652 proteins and 12,988 phosphosites were identified. Of them, 5322 proteins (with > 1 unique peptide) and 2162 phosphosites detected in > 50% of the samples were utilized for further data analysis (Figure S1E, F, and I–L). Additionally, inductively coupled plasma MS (ICP-MS) analysis of the Mg content was conducted on 115 paired samples, and correlation analysis of the QC samples confirmed the reliability of the measurements (Figure S1M). Whole-exome sequencing (WES) data were obtained from a previous study [38], in which 16,234 mutated genes with fewer than 5000 amino acids in 76 paired samples were reported.

Our findings showed that the intratumoral Mg content ranged from 162 to 920 μg per gram of extracted protein (Figure 1B). The Wilcoxon rank-sum test identified higher levels of Mg in tumors than in distant normal tissues (DNTs) (Figure 1C), and the levels of Mg were significantly different between the left- and right-sided tumors (Figure 1D and E). Our Kaplan–Meier survival analysis of 115 CRC patients revealed that lower levels of Mg in tumors were associated with poor overall survival (OS) (Figure 1F). The clinical characteristics of CRC patients according to Mg content are summarized in **Table 1**. Multivariate Cox regression models demonstrated that Mg content remained significantly associated with CRC survival, even after adjusting for confounding prognostic factors, such as renal function and liver function (Figure S2A–I; Table 1), indicating that Mg content serves as an independent predictor of survival. Moreover, we conducted an independent analysis to investigate the relationship between Mg levels and prognosis in left-sided and right-sided CRC patients. Interestingly, we observed a notable connection between Mg levels and the prognosis of CRC patients, specifically in right-sided cases, while a less significant association was found in left-sided cases (Figure 1G and H; Table 1). Additionally, no significant variation in Mg content was observed between tumors from female and male patients (Figure 1I; Table 1). Therefore, the following correlation analysis between Mg and omics data did not consider the influence of gender.

**Table 1:** Clinical characteristics of CRC patients according to Mg content

Characteristic	All patients (*n* = 115)	Low-Mg group (*n* = 36)	High-Mg group (*n* = 79)
Gender			
Female	49 (42.61%)	13 (36.11%)	36 (45.57%)
Male	66 (57.39%)	23 (63.89%)	43 (54.43%)
Mean age ± SD (year)	52.30 ± 16.38	53.19 ± 15.48	51.90 ± 16.76
Primary site			
Colon	86 (74.78%)	30 (83.33%)	56 (70.89%)
Rectum	29 (25.22%)	6 (16.67%)	23 (29.11%)
Tumor region			
Right-sided	49 (47.12%)	23 (67.65%)	26 (37.14%)
Left-sided	55 (52.88%)	11 (32.35%)	44 (62.86%)
TNM stage			
I	12 (10.62%)	1 (2.78%)	11 (14.29%)
II	34 (30.09%)	14 (38.89%)	20 (25.97%)
III	57 (50.44%)	16 (44.44%)	41 (53.25%)
IV	10 (8.85%)	5 (13.89%)	5 (6.49%)
OS status			
0: Living	61 (53.04%)	11 (30.56%)	50 (63.29%)
1: Deceased	54 (46.96%)	25 (69.44%)	29 (36.71%)
Mean OS ± SD (month)	60.65 ± 34.74	49.20 ± 35.17	65.86 ± 33.27
Lynch			
Lynch	0 (0%)	0 (0%)	0 (0%)
Non-Lynch	115 (100%)	36 (100%)	79 (100%)
Response to chemotherapy			
PR/SD	46 (61.33%)	12 (44.44%)	34 (70.83%)
PD	29 (38.67%)	15 (55.56%)	14 (29.17%)
Mean Mg content ± SD (Mg/protein, × 10^−3^ μg/g)	490,545.58 ± 136,078.44	344,642.11 ± 63,635.62	557,033.23 ± 104,827.95
Mean albumin ± SD (g/l)	40.44 ± 4.64	39.75 ± 4.92	40.76 ± 4.46
Mean ALT ± SD (U/l)	22.31 ± 23.71	23.92 ± 33.42	21.56 ± 17.45
Mean GGT ± SD (U/l)	37.74 ± 130.07	54.67 ± 215.50	29.92 ± 55.67
Mean creatinine ± SD (μM)	75.48 ± 19.87	76.59 ± 18.51	74.97 ± 20.44
Mean IDIL ± SD (μM)	7.06 ± 3.00	7.01 ± 3.01	7.08 ± 3.00
Mean AST ± SD (U/l)	21.84 ± 14.36	22.31 ± 20.92	21.63 ± 9.97
Mean urea ± SD (mM)	5.37 ± 1.60	5.13 ± 1.49	5.48 ± 1.63
Mean uric acid ± SD (μM)	314.13 ± 94.45	308.69 ± 97.86	316.64 ± 92.73
Mean DBIL ± SD (μM)	3.74 ± 2.59	3.93 ± 2.20	3.66 ± 2.75
Mean TBIL ± SD (μM)	10.80 ± 5.24	10.94 ± 4.60	10.74 ± 5.50
Family history			
CRC history	3 (2.61%)	1 (2.78%)	2 (2.53%)
Unknown	112 (97.39%)	35 (97.22%)	77 (97.47%)

*Note*: Values are presented as No. (%) unless otherwise indicated. CRC, colorectal cancer; TNM, tumor node metastasis; SD, standard deviation; OS, overall survival; PR, partial response; SD, stable disease; PD, progressive disease; ALT, alanine transaminase; GGT, γ-glutamyltransferase; IDIL, indirect bilirubin; AST, aspartate transaminase; DBIL, direct bilirubin; TBIL, total bilirubin.

### Low Mg content in tumors is linked to genome instability

**Figure 2:**
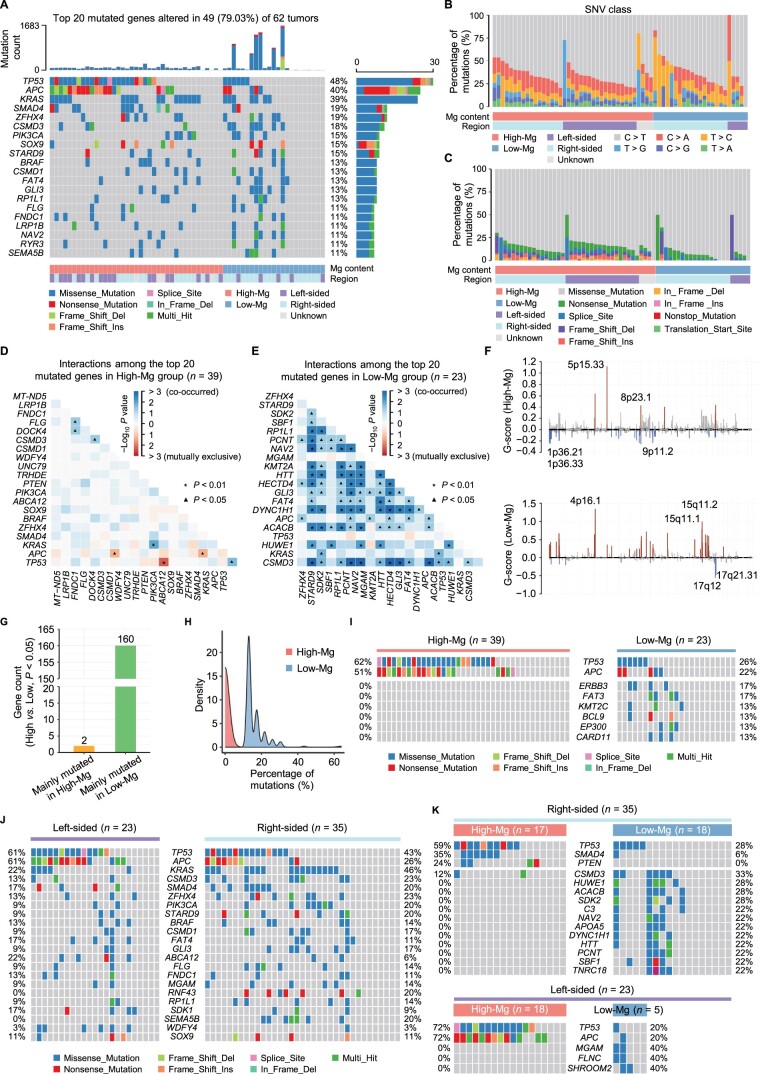
Low Mg content in tumors is linked to genome instability **A**. Genetic profile of the top 20 mutated genes in High-Mg and Low-Mg tumors. Somatic mutations in the top 20 genes were observed in 49 of 62 tumors. The top bar plot illustrates the cumulative count of somatic mutations in each patient, while the right bar plot depicts the distribution and composition of mutation types for each gene. **B**. Percentages of SNVs in High-Mg and Low-Mg groups. **C**. Percentages of the classes of mutations in High-Mg and Low-Mg groups. **D**. and **E**. The interactions among the top 20 mutated genes in High-Mg (D) and Low-Mg (E) groups. Fisher’s exact test (*, *P* < 0.01; ▲, *P* < 0.05). **F**. Focal peaks exhibiting significant SCNA (red) and deletion (blue) (GISTIC2 *q*-value < 0.1) are displayed for both the High-Mg and Low-Mg groups. The top 5 amplified and deleted cytobands are labeled. **G**. Comparison of genes with significant variations in mutation frequencies between the High-Mg and Low-Mg groups. Fisher’s exact test (*P* < 0.05). **H**. Density plot displaying the mutation frequencies of genes in the High-Mg and Low-Mg groups. **I**. Genetic profile of the CRC driver genes with significant differences in mutation frequency between the High-Mg and Low-Mg groups. Fisher’s exact test (*P* < 0.05). **J**. Comparison of gene mutation frequencies between the left- and right-sided tumors. Genes with mutations in more than 7 out of 62 patients are displayed. **K**. Comparison of gene mutation frequencies between the High-Mg and Low-Mg tumors on the left side (lower) or right side (upper). Genes with mutations in more than 5 out of 62 patients are displayed. SNV, single nucleotide variant; GISTIC, Genomic Identification of Significant Targets in Cancer; SCNA, somatic copy number alteration.

Mg is important for DNA stabilization, DNA replication, and DNA repair [39,40]. To investigate the impact of low Mg content on CRC at the genomic level, we analyzed Mg-associated genomic data. Of the 76 CRC patients analyzed by WES, 20 had hypermutation in tumors (> 10 mutations/Mb) (Table S2) [38]. To exclude hypermutations caused by mutated mismatch repair (MMR) genes and major replicase genes, including *MSH2*, *MSH6*, *MLH1*, *PSM2*, *POLD*, and *POLE*, we removed 14 hypermutated samples with mutations in these genes [38,41]. Statistical analysis revealed that *TP53* had the highest frequency of mutation (48%), followed by *APC* (40%) and *KRAS* (39%), in the remaining 62 CRC patients (**Figure 2**A). The total percentage of single nucleotide variants (SNVs) differed between the High-Mg (39 cases) and Low-Mg (23 cases) groups, with the most frequent thymine to cytosine (T > C) transition occurring in the Low-Mg group (Figure 2B). Additionally, the mutation types were different between the two groups, and more mutation types were observed in the High-Mg group (Figure 2C). Association analysis of the top 20 mutated genes in the two groups using the somatic interaction algorithm showed that the mutations in the High-Mg group were mutually exclusive, while the mutations in the Low-Mg group mainly co-occurred (Figure 2D and E), suggesting that low Mg content may cause simultaneous mutations in many genes, possibly due to increased genome instability and the dysregulation of DNA replication and repair processes [39,40,42–44]. In addition, somatic copy number alteration (SCNA) analysis revealed distinct patterns in the cytobands at the locations of the variant sites. In the High-Mg group, amplified regions were observed at 5p15.33 and 8p23.1, while in the Low-Mg group, amplified regions were found at 4p16.1, 15q11.1, and 15q11.2. Conversely, deleted regions were identified at 1p36.21, 1p36.33, and 9p11.2 in the High-Mg group and at 17q12 and 17q21.31 in the Low-Mg group (Figure 2F). Among the identified 7173 mutated genes with fewer than 5000 amino acids in 62 paired samples, the mutation frequencies of 162 genes were associated with Mg content, of which 160 genes exhibited higher mutation frequencies in the Low-Mg group (Figure 2G and H; Table S2), including six known CRC driver genes, *KMT2C*, *BCL9*, *ERBB3*, *EP300*, *FAT3*, and *CARD11* (Table S2) [45]. To our surprise, the mutation frequencies of *TP53* and *APC* were notably higher in the High-Mg group (Figure 2I).

Next, after excluding four tumors with unknown locations, we analyzed the impact of low Mg content on the genomic landscape of left-sided and right-sided CRC using the remaining 58 cases. Initially, we investigated the frequency of gene mutations in CRC and observed that the mutation frequencies of *TP53* and *APC* were higher on the left side, whereas *KRAS* exhibited a higher mutation frequency on the right side (Figure 2J), aligning with previous findings [29]. Subsequently, we explored the influence of Mg levels on gene mutations in left-sided and right-sided CRC separately. The results revealed that the majority of genes displayed higher mutation frequencies in the Low-Mg group on both sides. Notably, *TP53* demonstrated a higher mutation frequency in the High-Mg group for both left- and right-sided CRC, whereas *APC* exhibited a higher mutation frequency exclusively in the High-Mg group of left-sided CRC (72% cases in High-Mg group *vs*. 20% cases in Low-Mg group) but not right-sided CRC (Figure 2K). Collectively, these results indicate that low Mg content in tumors probably increases the number of mutations in genes associated with CRC progression rather than initiation.

### Low Mg content in tumors is associated with EMT activation

**Figure 3:**
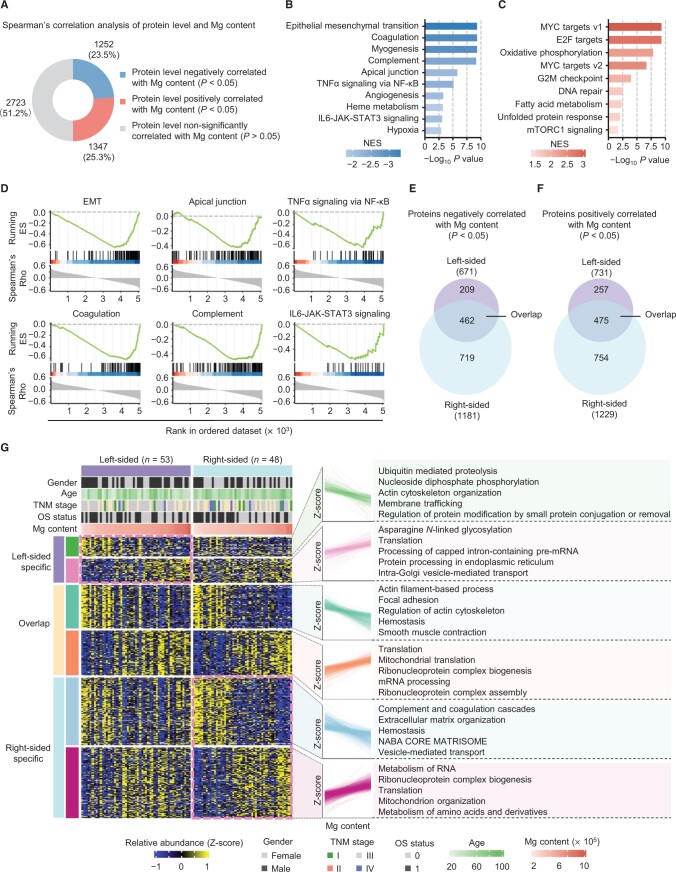
Low Mg content in tumors is associated with EMT activation **A**. Spearman’s rank correlation analysis of the relationship between protein levels and Mg content based on the 5322 proteins from GSEA pre-ranked hallmark analysis. Spearman’s rank correlation test (*P* < 0.05). **B**. and **C**. Bar plots showing the pathways negatively (B) and positively (C) associated with Mg content. **D**. Hallmark pathways related to immunity and metastasis. **E**. and **F**. Venn diagrams displaying the overlapping proteins negatively (E) or positively (F) associated with Mg content between the left- and right-sided tumors. Spearman’s rank correlation test (*P* < 0.05). **G**. Heatmap showing the levels of Mg-related proteins in the left- and right-sided tumors. The correlation patterns of the proteins with Mg content in distinct modules are shown. Z-scores of protein levels were mapped according to the Mg content in the left- and right-sided tumors. The top 5 pathways most enriched in the Metascape database using Mg-related proteins are shown. The gender, age, TNM stage, OS status, and Mg content are annotated above the heatmap. GSEA, gene set enrichment analysis; NES, normalized enrichment score; ES, enrichment score; TNM, tumor node metastasis; EMT, epithelial–mesenchymal transition.

To further explore the potential roles of Mg in CRC, we screened the Mg-associated proteomes in tumors according to a previously described strategy [37]. Correlation analysis of the levels of 5322 proteins and Mg content in tumors revealed that 1347 proteins were positively correlated with Mg content and 1252 proteins were negatively correlated with Mg content (**Figure 3**A; Table S3). Pathway enrichment analysis indicated that the 1252 proteins negatively correlated with Mg content were related to cell adhesion-related pathways, while the 1347 proteins positively correlated with Mg content were associated with messenger RNA (mRNA) processing and translation pathways (Figure S3A–D). Further gene set enrichment analysis (GSEA) of 5322 proteins revealed that pathways negatively correlated with Mg content mainly included immune- and metastasis-related pathways (Figure 3B–D), such as the coagulation cascade, complement system, IL-6–JAK–STAT3 signaling, TNF-α signaling via NF-κB, EMT, and angiogenesis (Figure 3B and D). On the other hand, pathways positively correlated with Mg content were predominantly related to cell proliferation and the cell cycle (Figure 3C).

Additionally, we conducted separate screening of Mg-associated proteins in left-sided and right-sided CRC (Figure 3E and F) and performed pathway enrichment analysis of these proteins (Figure 3G). The results indicated that proteins negatively correlated with Mg content in either left-sided or right-sided CRC, or both sides, were primarily associated with cell adhesion-associated pathways, such as regulation of actin cytoskeleton, actin cytoskeleton organization, focal adhesion, actin filament-based process, and extracellular matrix organization (Figure 3G). Notably, the complement and coagulation cascades were enriched exclusively in right-sided CRC, suggesting that Mg has a more prominent influence on the inflammatory response on the right side (Figure 3G). As Mg has demonstrated variations between left-sided and right-sided CRC, we next asked whether Mg has an impact on different subtypes of CRC patients. To achieve this goal, we classified these patients into three subtypes utilizing the top 25% most variable proteins (Figure S4A–C). While the OS did not significantly differ among the three subtypes, further analysis indicated that subtype II had a lower OS rate and notably lower Mg content than subtypes I and III did (Figure S4D and E). The proteins up-regulated in subtype II (subtype II *vs*. non-subtype II) were predominantly enriched in migration-related pathways (Figure S4F). Consistent with prior findings, Mg content is closely correlated with the cell adhesion of CRC tumors.

**Figure 4:**
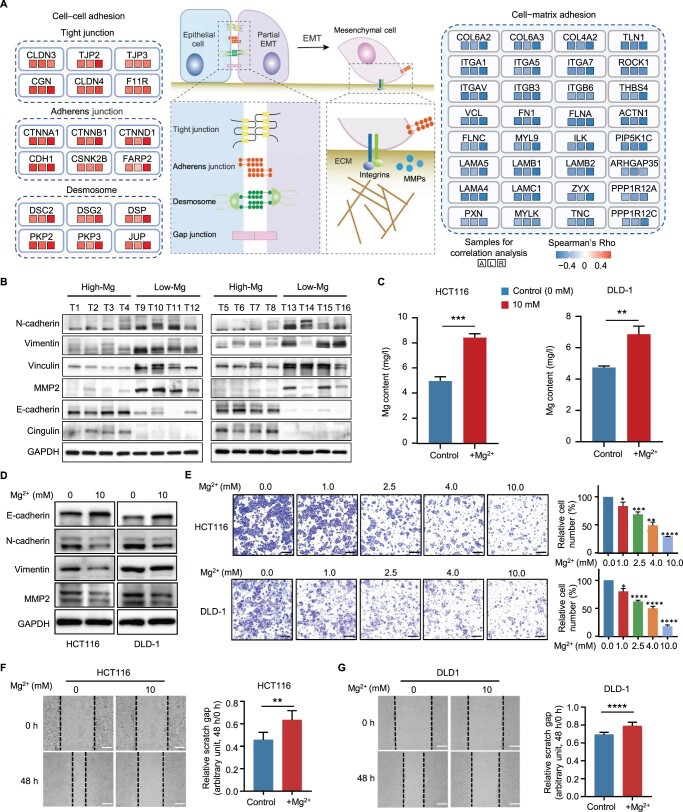
Functional validation of Mg in EMT activation in tumor cells **A**. Diagram showing a schematic of the EMT and cell adhesion pathways. Spearman’s correlation coefficients between Mg content and the levels of core components involved in cell–cell adhesion and cell–matrix adhesion are shown. A, L, and R indicate that the correlations were analyzed using all 104 tumor samples, 54 left-sided tumor samples, and 48 right-sided tumor samples, respectively. **B**. Immunoblots showing the expression of N-cadherin, vimentin, vinculin, MMP2, E-cadherin, and cingulin in High-Mg and Low-Mg tumors. **C**. Measurement of Mg content in HCT116 and DLD-1 cells treated with or without MgCl_2_ by ICP-MS. Student’s *t*-test (***, *P* < 0.001; **, *P* < 0.01). **D**. Western blotting analysis of vimentin, E-cadherin, N-cadherin, and MMP2 in colon cancer cell lines with (10 mM) or without MgCl_2_ treatment. **E**. Representative images (left) and quantification results (right) of the migration assays using colon cancer cells treated with increasing concentrations of MgCl_2_. Scale bars, 500 μm. Student’s *t*-test (****, *P* < 0.0001; ***, *P* < 0.001; **, *P* < 0.01; *, *P* < 0.05). **F**. and **G**. Representative images (left) and quantification results (right) of the wound healing assays using HCT116 (F) and DLD-1 (G) cells treated with (10 mM) or without MgCl_2_. Scale bars, 200 μm. Student’s *t*-test (****, *P* < 0.0001; **, *P* < 0.01). ICP-MS, inductively coupled plasma mass spectrometry; ECM, extracellular matrix; GAPDH, glyceraldehyde-3-phosphate dehydrogenase.

Considering the findings from GSEA (Figure 3B and C), it can be inferred that low Mg content in tumors potentially affects tumor metastasis through the regulation of cell adhesion-related pathways. Consistent with our assumption, many proteins linked to cell–cell adhesion, such as tight junction proteins CLDN3, TJP2, and CGN, adherens junction proteins CDH1, CTNNB1, and CTNND1, and desmosome proteins DSC2, DSG2, and JUP, showed significant positive correlations with Mg content. These proteins were crucial for maintaining the epithelial phenotype (**Figure 4**A; Table S4). In contrast, many cell–matrix adhesion proteins critical for maintaining the mesenchymal phenotype, such as VCL, ITGA1, ITGB3, FN1, and FGA, exhibited significant negative correlations with Mg content (Figure 4A). Further immunoblotting confirmed the increases in N-cadherin, vimentin, vinculin, and MMP2 and the decreases in E-cadherin and cingulin in Low-Mg tumors (Figure 4B), indicating the potential role of Mg in the EMT process.

To validate the role of Mg in the regulation of tumor metastasis, Transwell and wound healing assays were conducted using HCT116 and DLD-1 cell lines. ICP-MS analysis confirmed the successful uptake of Mg^2+^ into colon cancer cell lines (Figure 4C). Immunoblot analysis revealed that treatment with magnesium chloride (MgCl_2_) increased E-cadherin expression and reduced vimentin, MMP2, and N-cadherin expression in HCT116 and DLD-1 cells (Figure 4D). In line with the changes observed in EMT markers, both the Transwell and wound healing assays demonstrated that treatment with MgCl_2_ significantly decreased the migratory ability of colon cancer cells (Figure 4E–G). Taken together, our findings suggest that low Mg content in tumors may activate EMT by disrupting the homeostasis of cell adhesion molecules.

### Potential mechanisms of EMT-related cell adhesion molecule alterations induced by low Mg content

Numerous studies have provided evidence indicating that inflammation is a key factor contributing to the loss of cell adhesion molecules [46–48]. Our proteomic analysis of Mg-associated proteins indicated that the expression of 44 complement and coagulation components, such as C1R, C1Q, C2, C3, and C5, was negatively correlated with Mg content and significantly increased in Low-Mg tumors and DNTs (Figure S3C) [49], suggesting the activation of the complement pathway in these tumors and paired DNTs [50], which is known to induce inflammation [51] and affect the expression of cell adhesion molecules (Figure S3D) [52–54].

**Figure 5:**
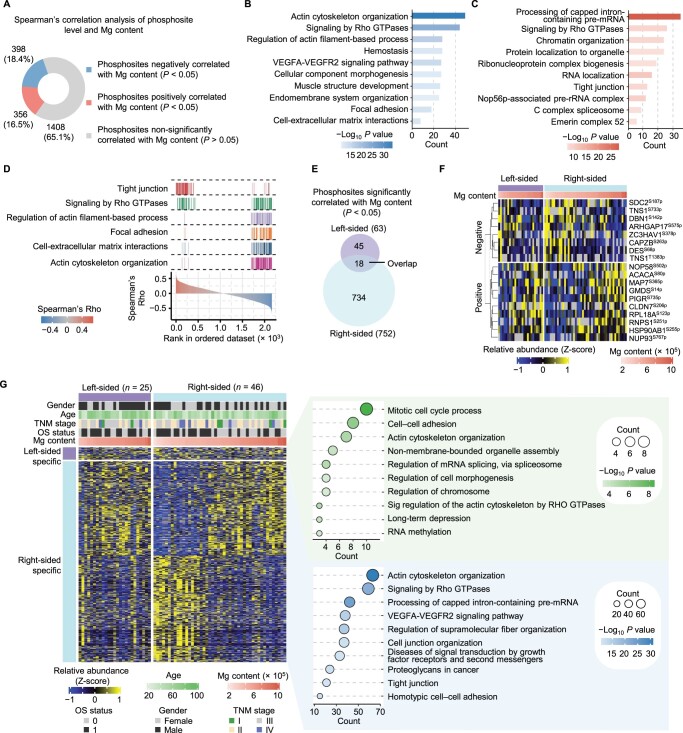
Impacts of Mg on the phosphoproteome of CRC **A**. Spearman’s rank correlation analysis of the relationship between the levels of phosphosites and Mg content. Spearman’s rank correlation test (*P* < 0.05). **B**. and **C**. Pathway enrichment analysis of the proteins containing Mg negatively (B) and positively (C) associated phosphosites using Metascape database. **D**. Mg-correlated pathways related to cell adhesion are shown with Spearman’s correlation coefficients obtained from (A). **E**. Venn diagrams displaying the overlapping phosphosites significantly correlated with Mg content between the left- and right-sided tumors. Spearman’s rank correlation test (*P* < 0.05). **F**. Heatmap displaying the levels of 18 common Mg-related phosphosites in left- and right-sided tumors. **G**. Heatmap showing the levels of phosphosites specifically correlated with Mg content in tumors on the left and right sides. The top 5 pathways enriched by Metascape based on the proteins with significantly Mg-correlated phosphosites are shown. Annotations above the heatmap include information such as gender, age, TNM stage, OS status, and Mg content. The heatmap illustrates the relative expression of phosphosites, utilizing the z-score for representation.

Additionally, previous studies have shown that changes in protein phosphorylation are important for the regulation of cell adhesion molecules [55,56]. Mg^2+^ is an important regulator of protein phosphorylation. When Mg is deficient, it not only reduces intracellular adenosine 5′-triphosphate (ATP) levels [30], but also impacts kinase and phosphatase activities [31,32], leading to alterations in phosphorylation signaling in cancer cells. Therefore, in addition to its impact on maintaining the homeostasis of cell adhesion molecules by regulating inflammation, it is conceivable that low Mg content may also modulate the levels of cell adhesion molecules through the modulation of phosphorylation in cancer cells. To investigate the impact of Mg on tumors through phosphorylation mechanisms, we analyzed the Mg-associated phosphoproteomes of CRC patients, and identified 356 and 398 phosphosites that were positively and negatively correlated with the Mg content in tumors, respectively (**Figure 5**A; Table S5). Enrichment analysis revealed that proteins corresponding to 398 negatively correlated phosphosites, such as ACTN4, DBN1, and VIM, were involved in actin cytoskeleton organization, signaling by Rho GTPases, regulation of actin filament-based process, focal adhesion and cell–matrix adhesion (Figure 5B), while proteins corresponding to 356 positively correlated phosphosites, such as CDK11B, CDK12, and THRAP3, exhibited significant enrichment in tight junction and signaling by Rho GTPases pathways (Figure 5C), indicating that the Mg-associated phosphoproteome in CRC is closely linked to EMT-associated pathways (Figure 5D).

Furthermore, we separately screened Mg-associated phosphosites in left-sided and right-sided CRC patients. The results revealed that 18 phosphosites were significantly correlated with Mg content in both left-sided and right-sided CRC, 45 phosphosites were only associated with Mg content in left-sided CRC, and 734 phosphosites were specifically related to Mg content in right-sided CRC (Figure 5E and F). Pathway enrichment analysis of the corresponding phosphorylated proteins unique to left-sided and right-sided CRC demonstrated that the Mg-associated phosphorylated proteins on both sides were associated with cell adhesion pathways, including cell–cell adhesion, tight junction, cell junction organization, and actin cytoskeleton organization pathways (Figure 5G). Furthermore, through an integrative analysis of both proteomic and phosphorylomic data, we identified 128 and 118 phosphosites positively and negatively correlated with Mg content, respectively. These correlations were observed independently of protein levels (Figure S5A–C). Furthermore, enrichment analysis of phosphoproteins exhibiting significant correlations with Mg content highlighted their associations with cell adhesion pathways, including actin cytoskeleton organization and cytoskeleton organization pathways (Figure S5D and E).

**Figure 6:**
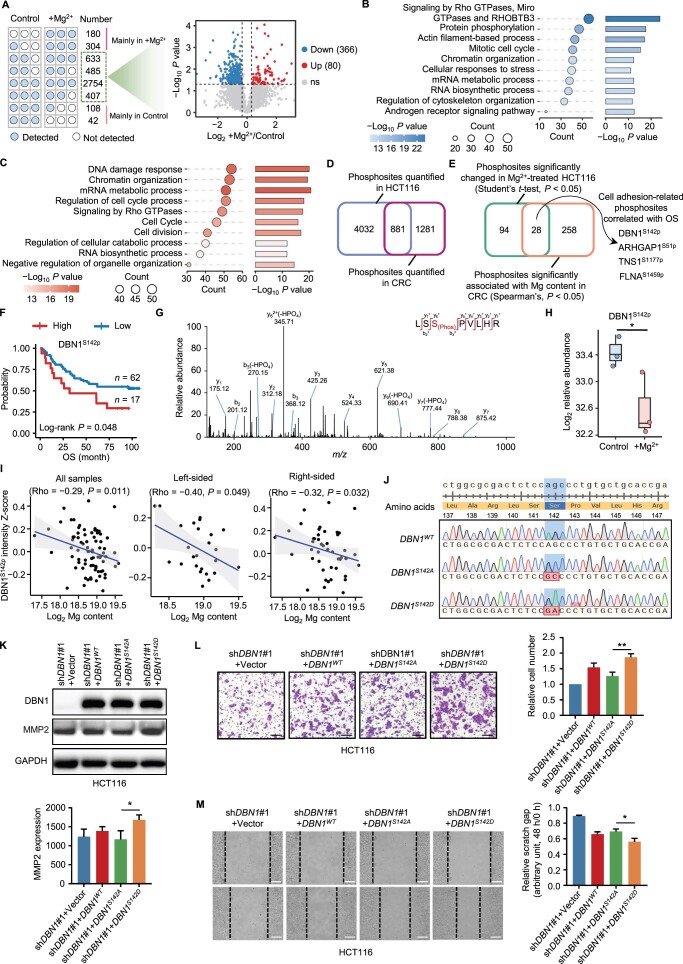
Mg-regulated DBN1^S142p^ reduction inhibits cell migration **A**. Left: number of phosphosites occurring in the +Mg^2+^ group or in the control group. The phosphosites mainly identified in the Mg^2+^ and control groups were considered up-regulated (484) and down-regulated (150), respectively. Right: volcano plot showing 80 up-regulated and 366 down-regulated phosphosites in the +Mg^2+^ group *vs.* in the control group. The volcano plot was constructed using phosphosites identified in at least two replicates of each group. The cutoff is ratio of +Mg^2+^/Control > 1.2 or < 0.83 and *P* < 0.05 (Student’s *t*-test). **B**. and **C**. Pathway enrichment analyses of the corresponding proteins with down-regulated (516) (B) and up-regulated (564) (C) phosphosites in the +Mg^2+^ group using Metascape. **D**. Venn diagram showing the number of overlapping phosphosites quantified in HCT116 cells and CRC patients. **E**. Venn diagram showing the number of overlapping phosphosites with significant changes in Mg^2+^-treated HCT116 cells and significant correlations with Mg content in CRC patients. **F**. Survival analysis of 79 CRC patients with different DBN1^S142p^ levels in tumors. Log-rank test (*P* < 0.05). **G**. MS/MS spectrum of the identified phosphopeptide containing DBN1^S142p^. **H**. Comparison of the DBN1^S142p^ levels in the control and +Mg^2+^ groups. Student’s *t*-test (*, *P* < 0.05). **I**. Spearman’s rank correlation analysis of DBN1^S142p^ and Mg content in all (72), left-sided (25), and right-sided (46) tumors. **J**. Mutation information for the *DBN1^WT^*, *DBN1^S142A^*, and *DBN1^S142D^* genes and sequencing results. **K**. Immunoblotting assays were used to determine the effects of DBN1 mutations on MMP2 expression. Student’s *t*-test (*, *P* < 0.05). **L**. and **M**. Transwell migration (L) and wound healing (M) assays were performed with cells transfected with vector, *DBN1^WT^*, *DBN1^S142A^*, or *DBN1^S142D^*. Scale bars, 500 μm (L) and 200 μm (M). Student’s *t*-test (**, *P* < 0.01; *, *P* < 0.05).

To validate the direct impacts of Mg on colon cancer cells via phosphorylation, we analyzed the phosphoproteomes of colon cancer cells treated with and without MgCl_2_. As a result, of the 15,235 identified phosphosites, 564 sites were increased in the MgCl_2_-treated group, and 516 sites were reduced (**Figure 6**A; Table S6). Notably, cell adhesion pathways such as actin filament-based processes, regulation of actin cytoskeleton organization, and signaling by Rho GTPases were enriched using proteins with differentially changed phosphosites (Figure 6B and C), consistent with the above phosphoproteomic data from CRC tissues (Figure 5B and C). Overlapping analysis indicated that the expression of 28 phosphosites significantly changed with Mg content in both HCT116 and CRC tissues (Figure 6D and E). Among them, four phosphosites, including DBN1^S142p^, ARHGAP1^S51p^, TNS1^S1177p^, and FLNA^S1459p^, were involved in cell adhesion (Figure 6E). Additionally, a high degree of phosphorylation at these four sites was found to be a predictor of unfavorable prognosis among CRC patients (Figure 6E and F, Figure S6A). Taken together, our results indicate that low Mg content in tumors can reshape the protein phosphorylation network of proteins associated with EMT and may ultimately affect tumor cell migration.

### Mg-regulated DBN1^S142p^ reduces the interaction between DBN1 and ACTN4 and contributes to EMT

**Figure 7:**
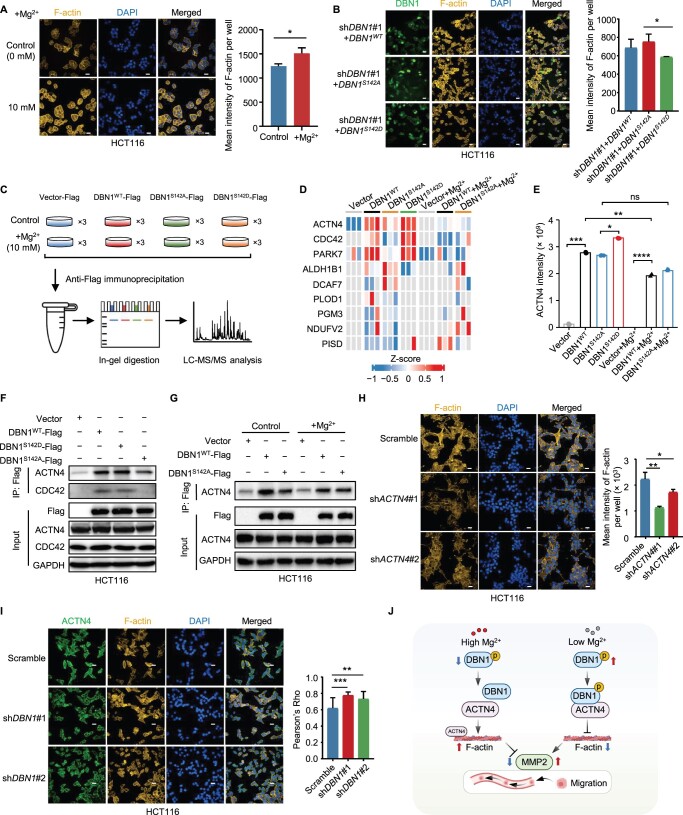
Mg-regulated DBN1^S142p^ reduces the interaction between DBN1 and ACTN4 and contributes to EMT **A**. Effect of MgCl_2_ treatment on the formation of F-actin determined by immunofluorescence assays in HCT116 cells. Scale bars, 500 μm. Student’s *t*-test (*, *P* < 0.05). **B**. Effects of DBN1^WT^, DBN1^S142A^, and DBN1^S142D^ on the formation of F-actin determined by immunofluorescence assays. Scale bars, 500 μm. Student’s *t*-test (*, *P* < 0.05). **C**. Schematic representation of the identification of DBN1-binding proteins affected by DBN1^S142p^ and Mg^2+^ through affinity purification followed by MS analysis. **D**. Heatmap showing nine DBN1-binding proteins affected by Mg^2+^ through regulating DBN1^S142p^. Z-scores of protein levels were used. **E**. The relative intensity of the DBN1-binding protein ACTN4 determined by MS in immunoprecipitation assays under different experimental conditions. Student’s *t*-test (****, *P* < 0.0001; ***, *P* < 0.001; **, *P* < 0.01; *, *P* < 0.05; ns, no significance). **F**. Immunoblotting assays showing the effects of DBN1^S142p^ on the interactions of ACTN4 and CDC42 with DBN1. **G**. Immunoblotting assays showing the effects of Mg on the interaction between ACTN4 and DBN1 affected by DBN1^S142p^. **H**. Effect of ACTN4 on the formation of F-actin determined by immunofluorescence assays. Scale bars, 500 μm. Student’s *t*-test (**, *P* < 0.01; *, *P* < 0.05). **I**. Co-localization of ACTN4 and F-actin was determined by immunofluorescence assays. Scale bars, 500 μm. Student’s *t*-test (***, *P* < 0.001; **, *P* < 0.01). **J**. Potential mechanism of Mg-regulated cell migration via modulation of DBN1^S142p^. LC, liquid chromatography; DAPI, 4′,6-diamidino-2-phenylindole.

DBN1 is a protein that binds to F-actin, and is essential for maintaining the levels of cell adhesion molecules [57,58]. However, the functions of DBN1^S142p^ in CRC and the mechanism by which Mg-regulated DBN1^S142p^ contributes to the EMT process are completely unknown. The confident identification of DBN1^S142p^ was verified through the MS/MS spectrum (Figure 6G). Statistical analysis revealed a significant decrease in DBN1^S142p^ in the MgCl_2_-treated group (Figure 6H). Moreover, a strong negative correlation was observed between DBN1^S142p^ and Mg content in CRC, irrespective of its sidedness (Figure 6I). To explore the role of DBN1^S142p^ in the regulation of EMT, we transfected wild-type *DBN1* or mutant *DBN1* (*DBN1^S142D^* or *DBN1^S142A^*) into *DBN1*-knockdown HCT116 cells that mimics the phosphorylation (DBN1^S142D^) or de-phosphorylation (DBN1^S142A^) state (Figure 6J). Given that DBN1 is an F-actin-binding protein and that the assembly of F-actin can promote MMP2 expression [57,58], we assumed that the Mg-regulated decrease in DBN1^S142p^ might serve as a signal for triggering F-actin disassembly and promoting MMP2 degradation. Immunoblotting revealed that, compared with DBN1^S142A^, DBN1^S142D^ remarkably up-regulated MMP2 (Figure 6K). Consistently, both Transwell and wound healing assays demonstrated that the DBN1^S142A^ mutation significantly inhibited cell migration (Figure 6L and M). Further studies showed that MgCl_2_ treatment, which was able to reduce the level of DBN1^S142p^ (Figure 6H), obviously enhanced the formation of F-actin (**Figure 7**A, Figure S6B). Consistent with these findings, DBN1^S142A^, which mimics the de-phosphorylation state, increased the formation of F-actin, compared to DBN1^S142D^ (Figure 7B). In addition, we explored the function of ARHGAP1^S51A^. Although the ARHGAP1^S51A^ mutation notably inhibited cell migration, it did not impact F-actin formation, in contrast to ARHGAP1^S51D^. This suggests that ARHGAP1^S51p^ inhibits cell migration through mechanisms distinct from F-actin formation (Figure S6C–E).

To elucidate the impact of Mg-regulated DBN1^S142p^ on F-actin polymerization, we employed immunoprecipitation coupled with MS analysis to identify proteins binding to DBN1, whose interactions are influenced by DBN1^S142p^ and Mg^2+^ (Figure 7C). As a result, we identified 376 proteins binding to DBN1, with 30 and 46 proteins being enriched in the DBN1^S142D^ and DBN1^S142A^ groups, respectively (Figure S7A and B). Notably, some of these proteins are involved in actin assembly and disassembly, suggesting that the level of DBN1^S142p^ may affect its binding to cytoskeleton-related proteins. Upon comparing the differential binding proteins between the DBN1^WT^ and DBN1^WT^+Mg^2+^ groups, we noted that 27 proteins exhibited increased interactions with DBN1, while 56 proteins showed diminished binding in response to MgCl_2_ treatment (Figure S7C). Of the 83 differentially expressed proteins, 73 demonstrated no notable changes between the DBN1^S142A^ and DBN1^S142A^+Mg^2+^ groups (Figure S7C), indicating that Mg^2+^ might modulate the interactions of these proteins with DBN1 by altering DBN1^S142p^. Among these 73 proteins, 53 whose interactions were weakened by Mg^2+^ were primarily associated with the actin cytoskeleton (Figure S7D), indicating that Mg^2+^ could affect the interaction between DBN1 and these cytoskeletal proteins, which may further impact the depolymerization of F-actin. Additional comparisons were performed to identify the proteins that bind to DBN1 in both the phosphorylated and de-phosphorylated states at the S142 residue (Figure S7E and F). The results showed that Mg suppressed DBN1^S142p^ to inhibit the interaction of three proteins, namely, ACTN4, CDC42, and PARK7 (Figure 7D and E, Figure S7E–G; Table S7), while it enhanced the interaction of six other proteins, namely, ALDH1B1, DCAF7, PLOD1, PGM3, NDUFV2, and PISD (Figure 7D, Figure S7F). Among these proteins, ACTN4 and CDC42 are well known for their close association with the cell cytoskeleton [59].

Previous studies have indicated that ACTN4 participates in the formation of F-actin and F-actin stability is compromised when ACTN4 is detached from F-actin [59]. Thus, we proposed that the interaction of DBN1 with ACTN4 results in the detachment of ACTN4 from F-actin. The de-phosphorylation of DBN1 at S142 could weaken the interaction between DBN1 and ACTN4, thereby promoting the interaction of ACTN4 with F-actin and enhancing F-actin stability. To investigate this possibility, we overexpressed the Flag-tagged vector, DBN1^WT^, DBN1^S142A^, and DBN1^S142D^ in *DBN1*-knockdown HCT116 cells and performed immunoprecipitation under identical experimental conditions. Immunoblotting confirmed the ability of ACTN4 to interact with DBN1 (Figure 7E). Moreover, the interaction between DBN1^S142A^ and ACTN4 was weakened, indicating that DBN1^S142p^ may affect the DBN1–ACTN4 interaction (Figure 7F).

To confirm that Mg^2+^ reduces the interaction between DBN1 and ACTN4 by decreasing the level of DBN1^S142p^, we constructed stable cell lines overexpressing the Flag-tagged vector, DBN1^WT^, and DBN1^S142A^. These cell lines were treated with or without MgCl_2_, followed by Flag-targeted immunoprecipitation assays. The immunoblotting results showed that MgCl_2_ treatment weakened the interaction between DBN1^WT^ and ACTN4, while the interaction between DBN1^S142A^ and ACTN4 was consistent after MgCl_2_ treatment (Figure 7G). These findings imply that Mg^2+^ may decrease the interaction between DBN1 and ACTN4 by reducing DBN1^S142p^. Then, we generated stable HCT116 cell lines with *ACTN4* knockdown and examined the effect of ACTN4 on F-actin polymerization (Figure S7H). *ACTN4* knockdown inhibited F-actin formation (Figure 7H). In contrast, the knockdown of *DBN1* resulted in a significant increase in the colocalization of ACTN4 with F-actin (Figure 7I), suggesting that the interaction between DBN1 and ACTN4 inhibits the formation of F-actin.

Collectively, these findings suggest that Mg^2+^ diminishes the interaction between DBN1 and ACTN4 by decreasing the level of DBN1^S142p^. This, in turn, enhances the binding of ACTN4 to F-actin and stabilizes F-actin, ultimately resulting in reduced MMP2 expression and decreased migratory ability in colon cancer cells (Figure 7J).

## Discussion

In this study, we presented, for the first time, an atlas of the Mg-associated genome, proteome, and phosphoproteome in CRC, and demonstrated that low Mg content in tumors promoted genome instability and tumor metastasis. This work has yielded several novel findings. First, our study revealed that a large number of genes in Low-Mg tumors exhibited higher mutation frequencies and co-occurred, including several driver genes associated with tumor progression. Conversely, High-Mg tumors displayed high mutation frequencies in well-known initiation driver genes of CRC, such as *TP53* and *APC*, indicating that Mg deficiency may primarily impact tumor progression rather than initiation at the genomic level. Second, our study showed that Low-Mg tumors activated EMT by disrupting the homeostasis of adhesion molecules in cancer cells. This disruption was attributed not only to activated complement pathway and elevated inflammation in Low-Mg tumors but also to changes in the phosphorylation signaling of proteins associated with cell adhesion. Third, we discovered a novel phosphosite, DBN1^S142p^, regulated by Mg^2+^. Our results showed that Mg^2+^ reduced the interaction between DBN1 and ACTN4 by decreasing DBN1^S142p^. This reduction in DBN1^S142p^ resulted in increased binding of ACTN4 to F-actin, thereby stabilizing F-actin and ultimately leading to reduced MMP2 expression and inhibition of colon cancer cell migration. Overall, this work uncovers the roles of Mg in CRC and demonstrates that low Mg content in tumors could be a potential driver factor of CRC.

Cancer cells require a large amount of Mg to support their glycolytic metabolism, ATP production, and protein synthesis, which are essential for sustaining rapid proliferation [60]. Insufficient Mg uptake not only slows cancer cell proliferation but also leads to genome instability [61], as demonstrated in this work. Mg deficiency is known to induce low-grade systemic inflammation and cause increased production of reactive oxygen species (ROS) and proinflammatory cytokines, such as IL-6, TNF-α, and IL-1β [8,62], leading to genomic instability. Moreover, Mg plays a crucial role in regulating DNA replication and repair processes [39,40,42–44], which may partially explain why a significant number of CRC driver genes, such as *KMT2C* and *ERBB3*, are mutated in tumors with low Mg content. Tumor initiation and progression are determined by the inactivation of the tumor suppressors *APC* and *TP53* and the activation of the oncogene *KRAS* [63]. Surprisingly, the mutation frequencies of these genes were significantly higher in tumors with high Mg content (High-Mg/Low-Mg frequencies: *APC*, 51%/22%, *P* = 0.032; *TP53*, 62%/26%, *P* = 0.0091; *KRAS*, 44%/30%, *P* = 0.42). However, the mechanism by which high Mg levels induce mutations in the *APC* and *TP53* genes remains to be investigated. In addition, the screening of Mg-related gene mutations in left- or right-sided colon cancer, or on both sides, requires validation in larger CRC cohorts.

EMT, characterized by loss of cell–cell junctions, disruption of cell–matrix attachments, and cytoskeleton remodeling, can increase the mobility of cells. Many signaling pathways, such as the TGF-β, Wnt, and Hippo pathways, are associated with the regulation of EMT [64]. Mg deficiency is known to cause inflammation by disturbing the coordination of the innate immune system and the adaptive immune response [14], leading to rapid loss of cell adhesion molecules [46–48]. Alternatively, phospho-signals, which serve as the first wave of response to intracellular and extracellular changes, extensively exist during the EMT process [65,66]. For example, phosphorylation of SNAIL [67], GSK3β [68], and EGFR [69] contributes to the regulation of EMT. Our findings, for the first time, systematically highlight the significance of Mg’s direct regulation of phosphorylation in cancer cells, which is critical for maintaining the homeostasis of cell adhesion molecules and is closely linked to the EMT process. Specifically, our experiments revealed that Mg^2+^ played a crucial role in reducing the interaction between DBN1 and ACTN4 by decreasing the level of DBN1^S142p^. This, in turn, had a significant impact on F-actin stability and the homeostasis of cell adhesion molecules. However, the exact mechanism by which Mg affects DBN1^S142p^ is not yet fully understood, although it may impact the activity of phosphatases, thereby influencing DBN1^S142p^. Additionally, the precise binding mode between DBN1 and ACNT4 remains elusive, and further biochemical experiments are necessary to determine how DBN1^S142p^ affects the interaction between DBN1 and ACNT4.

Adults typically consume approximately 330–350 mg of Mg daily. Previous studies have shown that cirrhotic patients with hepatocellular carcinoma (HCC) exhibit reduced serum Mg levels compared to those without HCC, independent of confounding factors such as dietary Mg intake and medications affecting Mg levels [70]. Moreover, mouse experiments have validated that the growth of primary tumors sequesters Mg from the extracellular environment, leading to hypomagnesemia [60]. Additionally, both our work and other studies have described a greater preference for Mg absorption in tumors than in normal tissues, which may increase the possibility of reducing serum Mg levels [60,70]. However, this speculation requires further evidence.

Previous clinical studies have demonstrated the potential benefits of Mg in the prevention and treatment of CRC. A population-based prospective study suggested that high Mg intake may reduce the risk of CRC in women [21]. Moreover, Mg supplementation can enhance the efficiency of drug treatment and minimize serious side effects in CRC patients. For example, the combination of 25(OH)D3 and Mg has been shown essential for reducing the risk of mortality in CRC patients [71]. Peripheral neuropathy is a common side effect caused by chemotherapy in CRC patients, but high dietary Mg intake can reduce its prevalence and severity [28]. Notably, in late-stage CRC patients treated with cetuximab [72] or bevacizumab [73], EGFR inhibition can result in Mg wasting due to decreased renal reabsorption, and a decrease in circulating Mg^2+^ may act as a predictive factor of treatment efficacy and outcome [74,75]. The extracellular matrix (ECM) is a complex network of proteins and other molecules that surround cells and play important roles in cell signaling, migration, and proliferation [76]. Abnormalities in ECM composition and organization are often associated with cancer progression and metastasis. ECM proteins themselves and ECM protein-interacting proteins such as integrins are known targets of cancer treatment [77,78]. Interestingly, by referring to known drug targets of Food and Drug Administration (FDA)-approved drugs or candidate drugs in clinical trials, we identified 22 clinically actionable cell–matrix proteins, such as COL3A1, FGA, and ITGA5, whose expression was negatively correlated with the Mg content in tumors (Spearman’s rank correlation test, *P* < 0.05) (Figure S8; Table S8), suggesting that Mg might serve as an adjuvant drug for precise CRC treatment by affecting the levels of these potential drug targets. Our research revealed that the functions of Mg are expected to promote the application of Mg reagents in the prevention and treatment of CRC.

## Materials and methods

### Sample collection and preparation

A total of 115 paired tumor samples and corresponding DNTs were obtained from treatment-naive CRC patients at West China Hospital of Sichuan University, Chengdu, China. The samples were rapidly snap-frozen in liquid nitrogen and then stored at −80°C for long-term preservation. This study was approved by the Research Ethics Committee of Biology Research, West China Hospital at Sichuan University [Approval No. 2020 (374)]. Informed consent and approval were obtained from all patients, and the results were reviewed accordingly. Detailed clinical information, such as age, gender, tumor region, OS status, OS (month), and tumor node metastasis (TNM) stage, is systematically recorded in Table S1. Patient follow-up was conducted over a median period of 67.25 months. OS was defined as the duration from surgery to either the patient’s death or the last follow-up visit. We used genomic, proteomic, and phosphoproteomic data from these CRC patients to elucidate the impact of Mg deficiency on CRC. Notably, the genomic, proteomic, and phosphoproteomic data for each patient were generated using the same sample.

### Proteomic and phosphoproteomic analyses

The protein extraction and digestion procedures were as follows. Tissues were homogenized and lysed using gentleMACS Dissociators (Catalog No. 130-093-235, Miltenyi Biotec, Nordrhein-Westfalen, Germany) with radioimmunoprecipitation assay lysis buffer (RIPA) buffer (Catalog No. P0013C, Beyotime, Shanghai, China). Cell samples were directly lysed in RIPA buffer. Subsequently, the prepared lysates were sonicated for 5 min at 227.5 W, with a 3-s on/10-s off cycle. After centrifugation at 20,000 *g* for 20 min at 4°C, the supernatant was transferred to a new tube, and Bradford protein assay was used to measure the protein concentration. For each sample, 100 μg of protein lysates was reduced using 10 mM tris (2-carboxyethyl) phosphine (TCEP) at 56°C for 60 min. Subsequently, the proteins were alkylated with 20 mM iodoacetamide for 30 min in the dark at 25°C. The protein samples were then precipitated using CH_3_OH, CHCl_3_, and H_2_O (CH_3_OH:CHCl_3_:H_2_O = 4:1:3, v/v). Finally, the proteins were digested with trypsin at a 1:50 ratio (trypsin/protein, w/w) for 12 h.

For TMT 10 labeling of peptides, TMT 10-plex Isobaric Label Reagent (Catalog No. 90110, Thermo Fisher Scientific, Waltham, MA) was dissolved in anhydrous acetonitrile (ACN) after reaching room temperature. Then, 10 μg and 40 μg of digested peptides from each sample for proteomic and phosphoproteomic analyses, respectively, were labeled with TMT reagents following the manufacturer’s instructions. Subsequently, the TMT-labeled peptides were combined and dried.

TMT-labeled peptide fractionation was performed as follows. Reversed-phase high-performance liquid chromatography (RP-HPLC) was used to fractionate peptides with a basic mobile phase for proteomics. The separation was carried out at a flow rate of 1 ml/min using a mixture of buffer A (98% H_2_O, 2% ACN, pH 10) and buffer B (90% ACN, 10% H_2_O, pH 10). The liquid chromatography (LC) gradient run spanned 120 min and followed this pattern: 3%–35% buffer B for 95 min, 35%–60% buffer B for 10 min, and 60%–100% buffer B for 15 min. The eluates were collected in 120 fractions, which were subsequently merged into 15 fractions for each batch of CRC samples. These fractions were then dried using a vacuum centrifuge. Following desalting with C18 ZipTips, the TMT-labeled peptides were subjected to LC-MS/MS analysis. For phosphoproteomic analysis, the TMT-labeled peptides from tissue samples were initially fractionated into 15 fractions using C18 solid-phase extraction (SPE) columns (100 mg/1 ml). These fractions were subsequently combined into 5 fractions and dried. In the case of cell phosphoproteomics, each sample was divided into 9 fractions using C18 SPE columns, which were then merged into 3 fractions prior to drying.

Phosphorylated peptides were enriched using PureCube Fe-NTA Agarose Beads (Catalog No. 31403-Fe, Cube Biotech, Monheim, Germany) according to the manufacturer’s instructions. The peptides were dissolved in 300 µl of loading buffer [85% ACN and 0.1% trifluoroacetic acid (TFA)] and then incubated with the prepared beads for about 60 min on a three-dimensional shaker at room temperature. Subsequently, the agarose beads were washed 4 times with washing buffer (80% ACN and 0.1% TFA) and then eluted with 150 µl of elution buffer (40% ACN and 15% ammonium hydroxide). To neutralize the eluate, 8 µl of 20% TFA was added. The elution buffer was subsequently dried under vacuum, and subjected to LC-MS/MS analysis after desalting using C18 ZipTips.

For proteomic analysis, LC-MS/MS analysis was conducted using an EASY-nLC 1200 system LC instrument coupled with an Orbitrap Exploris 480 mass spectrometer (Catalog No. BRE725539, Thermo Fisher Scientific). After desalting with ZipTip columns, the samples were dried and reconstituted in buffer A [98% H_2_O, 2% ACN, 0.1% formic acid (FA)]. The peptides were then loaded onto an in-house pulled and packed analytical column (75 μm × 30 cm) packed with C18 particles. Samples were analyzed using a 65-min gradient of 4% to 100% buffer B (0.1% FA in 80% ACN) at a flow rate of 300 nl/min in positive ion mode. The MS1 full scans (*m/z* 350–1800) were acquired with a resolution of 60,000. The automatic gain control (AGC) value was set to 300%, and the maximum injection time (MIT) was 50 ms. For MS/MS scans, the top 20 most abundant parent ions were selected under an isolation window of 0.7 *m/z*, and fragmentation was performed using a normalized collision energy (NCE) of 36%. The normalized AGC for MS/MS was set to 75%, and the MIT was 80 ms. Precursor ions with charge states of z = 1, 8, or unassigned charge states were excluded from further fragmentation.

For phosphoproteomic analysis, LC-MS/MS analysis was conducted using an EASY-nLC 1200 system LC instrument coupled with a Q Exactive HF-X high-resolution mass spectrometer (Catalog No. 0726042, Thermo Fisher Scientific). After desalting with ZipTip columns, the samples were dried and reconstituted in buffer A, consisting of 2% ACN and 0.1% FA. The peptides were then separated by a homemade trap column (2.5 cm × 75 μm) packed with Spursil C18 particles and an analytic column (25 cm × 75 μm) packed with ReproSil-Pur C18-AQ particles. Samples were analyzed using a 65-min gradient of 6% to 100% buffer B (0.1% FA in 80% ACN) at a flow rate of 330 nl/min in positive ion mode. The MS1 full scans (*m/z* 350–1600) were acquired with a resolution of 60,000. The AGC value was set to 3E6, and the MIT was set to 20 ms. For MS/MS scans, the top 20 most abundant parent ions were selected under an isolation window of 0.6 *m/z*, and fragmentation was performed using stepped NCEs of 25% and 31%. Precursor ions with charge states of z = 1, 8, or unassigned charge states were excluded from further fragmentation. For label-free cell phosphoproteomics, samples were analyzed using a 90-min gradient of 12% to 100% buffer B (0.1% FA in 80% ACN) at a flow rate of 330 nl/min in positive ion mode. The MS1 full scans (*m/z* 350–1800) were acquired with a resolution of 60,000. The AGC value was set to 3E6, and the MIT was set to 20 ms. For MS/MS scans, the top 20 most abundant parent ions were selected under an isolation window of 1.6 *m/z*, and fragmentation was performed using stepped NCEs of 25% and 27%. Precursor ions with charge states of z = 1, 8, or unassigned charge states were excluded from further fragmentation.

### MS database searching

All MS raw proteomic and phosphoproteomic data files were analyzed using MaxQuant (v1.6) and aligned against the Swiss-Prot human protein sequence database comprising 20,413 entries (updated 04/2019). For MS2 reporter ion quantification, the reporter mass tolerance was set at 0.02 Da. The peptide mass tolerance was set at 10 ppm, and only peptides and proteins with a false discovery rate (FDR) lower than 1% were kept for further data processing. Up to 2 missing cleavage sites were allowed. Cysteine carbamidomethylation was specified as a fixed modification, while oxidation of methionine and protein N-terminal acetylation were considered variable modifications. For phosphoproteomic analysis, phosphorylation (+ 79.9663 Da) of serine, threonine, and tyrosine residues were also added to the abovementioned variable modifications.

### Proteomic data cleaning

R (v4.2.1) was used to process the proteomic data to minimize systematic errors based on the “peptides.txt” from the MaxQuant output. Several preprocessing steps were performed to refine the data. First, potential contaminants and reverse proteins were excluded. Subsequently, only proteins with ≥ 2 unique peptides were selected for further analysis. To ensure comparability across samples within the same batch, the total protein abundance of each sample was adjusted to an equal level. To reduce the impact of noise, the protein intensity values of the tumor or DNT samples were divided by the protein intensity of an IS sample, yielding protein sample-to-standard (S/S) values. This step helped normalize the data and mitigate potential confounding factors. All the data from the 27 batches were combined into a matrix, with the samples represented as columns and proteins represented as rows. Additionally, the normalized values underwent an additional log_2_-transformation to facilitate subsequent analyses. The values in the matrix were transformed to column z-scores and row z-scores to normalize the data distribution across samples and proteins, respectively. Any values that were originally zero were considered missing and replaced with “NA”. To ensure data quality, proteins with > 50% missing values were excluded from the dataset, resulting in a refined and reliable set of proteins for further analysis.

### Phosphoproteomic data cleaning

R (v4.2.1) was used to process the phosphoproteome data to minimize systematic errors based on the “Phospho (STY) Sites.txt”. Several preprocessing steps were performed to refine the data. First, potential contaminants and reverse peptides were excluded. In addition, phosphopeptides with a localization probability > 0.75 were kept. To ensure comparability across samples within the same batch, the total intensities of phosphopeptides in each sample were adjusted to an equal level. To reduce the impact of noise, the phosphopeptide intensities of the tumors or DNT samples were divided by the phosphopeptide intensity of an IS sample, resulting in sample-to-standard (S/S) values. This step helped normalize the data and mitigate potential confounding factors. All the data from the 20 batches were combined into a matrix, with the samples represented as columns and phosphopeptides represented as rows. Additionally, the normalized values underwent an additional log_2_-transformation to facilitate subsequent analyses. The values in the matrix were transformed to column z-scores and row z-scores to normalize the data distribution across samples and phosphopeptides, respectively. Any values that were originally zero were considered missing and replaced with “NA”. To ensure data quality, phosphopeptides with > 50% missing values were excluded from the dataset.

### Measurement of Mg content in tissues

Tissue samples were homogenized and lysed using gentleMACS Dissociators (Catalog No. 130-093-235, Miltenyi Biotec) with RIPA buffer. Subsequently, the tissue lysates were sonicated for 5 min at 227.5 W, with a 3-s on/10-s off cycle. A Bradford protein assay was used to measure the protein concentration, and 350 μl of each sample was taken and used for the measurement of Mg content. The Mg content in each tissue was quantified by normalization to the protein concentration. The lyophilized samples were then treated with a suitable amount of 65% nitric acid (HNO_3_) overnight at room temperature. This process was continued by heating the samples in a heating block at 90°C for about 20 min. Subsequently, an equivalent volume of 30% H_2_O_2_ was added to each sample. The reaction was stopped after an additional 30 min, followed by a further heating step at 70°C for 15 min. The mean reduced volume was established, and subsequently, the samples were diluted with 1% HNO_3_. Measurements were conducted using an Agilent 7700 series ICP-MS instrument (Catalog No. Agilent 7700 ICP-MS, Agilent Technologies, Santa Clara, CA) under standard multi-element operating conditions employing a helium reaction gas cell. Calibration of the instrument was performed using certified multi-element ICP-MS standard calibration solutions with concentrations spanning 0, 5, 10, 50, 100, and 500 ppb for various elements. Moreover, a certified IS solution containing 200 ppb yttrium was employed as an internal control.

### WES and data processing

In our previous study, we utilized a total of 76 paired tumors and corresponding DNTs from CRC patients for WES analysis [35]. The detailed methods have been described previously. Briefly, genomic DNA was quantified by a Qubit DNA Assay Kit in a Qubit 2.0 Fluorometer (Catalog No. Q32866, Thermo Fisher Scientific). For each sample, 0.6 µg of genomic DNA was used as input material for sample preparation. Subsequently, WES libraries were prepared and captured using an Agilent SureSelect Human All Exon Kit (Catalog No. 5191-5735, Agilent Technologies). The DNA library, featuring 150 bp paired-end reads, was sequenced using an Illumina NovaSeq 6000 System (Catalog No. 20012850, Illumina, San Diego, CA). The initial fluorescence image files acquired from the HiSeq platform were converted to raw data through base calling and further transformed into the fast quality score (FASTQ) format. This format includes both sequence information and corresponding sequencing quality details.

### SCNA analysis

The exome sequencing data were aligned to the human genome hg19, and copy number variations (CNVs) were identified from the Binary Alignment/Map (BAM) files derived from WES for SCNA analysis. To integrate the results obtained from individual patients and identify recurrently amplified or deleted focal genomic regions in our samples, we employed Genomic Identification of Significant Targets in Cancer (GISTIC) 2.0 software (https://cloud.genepattern.org) [79]. Additionally, the R package “maftools” was used to display Q values less than 0.1. Moreover, to minimize false positives, the related parameters were set as follows: refgene file = Human_Hg19.mat, focal length cutoff = 0.50, gene gistic = yes, confidence level = 0.99. All the other parameters were maintained at their default settings.

### Differential abundance analysis

The cleaned proteomic and phosphoproteomic data were subjected to differential abundance analysis between tumors and DNTs through the Wilcoxon rank-sum test. Variables that contained > 50% missing values were excluded. *P* < 0.05 was considered to indicate statistical significance, and the fold change (FC) was calculated as the median log_2_ FC. Differential proteins and phosphosites in cell lines were detected through Student’s *t*-test. *P* < 0.05 was considered to indicate statistical significance, and FC was calculated as the median log_2_ FC. Pathway enrichment analysis was performed based on the Database for Annotation, Visualization and Integrated Discovery (DAVID) bioinformatics resources (https://david.ncifcrf.gov/) [80,81] and the Metascape database (http://metascape.org) [82].

### Correlation analysis

Pearson’s correlation analysis was performed between IS and QC samples to assess the quality of the proteomic and phosphoproteomic data. The correlations between Mg content and the levels of proteins or phosphosites were calculated via Spearman’s rank correlation analysis.

### Univariate survival analysis

The optimal cutoff point for the selected samples was calculated by the R package “survminer”. To assess differences between the categorical variables, the log-rank test was applied, and two-tailed tests and *P* < 0.05 were used for significance evaluation. In the R package “survminer”, survival curves were created through the Kaplan–Meier method for specific variables of interest. The estimation of hazard ratios (HRs) and 95% confidence intervals (CIs) was performed using the “coxph” function of the R package “survival”.

### Western blotting analysis

Proteins were extracted from tissues and cells with RIPA buffer and then quantified by the Bradford assay. Sodium dodecyl sulfate-polyacrylamide gel electrophoresis (SDS-PAGE) (10%) was used to separate the protein samples, which were subsequently transferred to polyvinylidene fluoride (PVDF) membranes (Catalog No. ISEQ00010, Millipore, Darmstadt, Germany). Next, the PVDF membranes were blocked with 5% milk in phosphate buffered saline with Tween 20 (PBST) and incubated overnight at 4°C with the following antibodies: anti-E-cadherin antibody (1:1000; Catalog No. 20874-1-AP, Proteintech, Wuhan, China), anti-N-cadherin antibody (1:1000; Catalog No. 22018-1-AP, Proteintech), anti-vimentin antibody (1:1000; Catalog No. 10366-1-AP, Proteintech), anti-vinculin antibody (1:1000; Catalog No. 66305-1-Ig, Proteintech), anti-MMP2 antibody (1:1000; Catalog No. 10373-2-AP, Proteintech), anti-cingulin antibody (1:1000; Catalog No. 21369-1-AP, Proteintech), anti-DBN1 antibody (1:1000; Catalog No. 10260-1-AP, Proteintech), anti-ACTN4 antibody (1:1000; Catalog No. 19096-1-AP, Proteintech), and anti-GAPDH antibody (1:5000; Catalog No. 60004-1-Ig, Proteintech). After overnight incubation, the membranes were washed three times with PBST, followed by incubation with the appropriate secondary antibody at room temperature for 1 h. Finally, the membrane was subjected to chemiluminescent detection using Immobilon Western HRP Substrate (Catalog No. WBKLS0500, Millipore).

### Cell culture and generation of stable cell lines

The HCT116 and DLD-1 human CRC cell lines were obtained from the Cell Bank/Stem Cell Bank, Chinese Academy of Sciences, and cultured in Dulbecco’s Modified Eagle Medium (DMEM; Catalog No. C11995-065, Gibco, Grand Island, NY) and Roswell Park Memorial Institute (RPMI) 1640 (Catalog No. 10270-106, Gibco) supplemented with 10% fetal bovine serum (FBS; Catalog No. FCS500, ExCell, Shanghai, China), 100 U of penicillin, and 100 μg/ml streptomycin (Catalog No. 15140-122, Gibco), respectively.

To construct DBN1-GFP-tagged and DBN1-Flag-tagged cells, the cDNA of *DBN1* was inserted into the pCDH-LGFP and pCDH-3×Flag vectors, respectively. The pLKO.1 vector was used for *DBN1* and *ACTN4* knockdown. To generate cells overexpressing *DBN1* or cells with silenced *DBN1* and *ACTN4*, a co-transfection approach was employed. HEK293T cells were co-transfected with psPAX2, pMD2.G, and either pCDH-LGFP-DBN1, pCDH-3×Flag-DBN1, sh*DBN1*, sh*ACTN4*, or their respective control plasmids. After 48 h, the medium containing the virus was collected and filtered. To enhance the transfection efficiency, 10 mg/ml polybrene (Catalog No. S2667, Sigma, Saint Louis, MI) was added. Subsequently, the cells were infected and selected with 1 μg/ml puromycin (Catalog No. ST551, Beyotime) for 48 h. The primers for *DBN1* and *ACTN4* knockdown were as follows: sh*DBN1*#1 (5′-CCGGCTGTGGAAATGAAGCGGATTACTCGAGTAATCCGCTTCATTTCCACAGTTTTTG-3′), sh*DBN1*#2 (5′-CCGGCCTGTGTTCTACAACAAGCCTCTCGAGAGGCTTGTTGTAGAACACAGGTTTTTG-3′), sh*ACTN4*#1 (5′-CCGGCATCGCTTCCTTCAAGGTCTTCTCGAGAAGACCTTGAAGGAAGCGATGTTTTTG-3′), and sh*ACTN4*#2 (5′-CCGGCCTGTCACCAACCTGAACAATCTCGAGATTGTTCAGGTTGGTGACAGGTTTTTG-3′). The primers for *DBN1* overexpression were *DBN1-PCDH-GFP* (5′-GATTCTAGAGCTAGCGAATTCATGGCCGGCGTCAGCTTCAGC-3′) and *DBN1-PCDH-Flag* (5′-GATGACAAGTCTAGAGAATTCATGGCCGGCGTCAGCTTCAGC-3′). The primers for *DBN1* mutations were *DBN1^S142A^* (5′-CGCGACTCTCCGCCCCTGTGCTGCA-3′) and *DBN1^S142D^* (5′-CGCGACTCTCCGACCCTGTGCTGCA-3′).

### Cell migration assay

For cell migration assay, 8.0-mm, 24-well plate chamber inserts were used (Catalog No. 354578, Corning Life Sciences, Corning, NY). A total of 3 × 10^5^ cells suspended in 200 μl of serum-free medium were added to the upper chamber of the inserts, while 800 μl of 10% FBS medium was added to the bottom chamber. After 24 h incubation, the cells were fixed with 4% paraformaldehyde (PFA) for 15 min and stained with 0.5% crystal violet for another 15 min. Cells on the upper surface of the inserts were retained, while cells on the underside were gently removed using a cotton swab. The captured images were analyzed using ImageJ software to quantify the number of cells.

### Wound healing assay

HCT116 and DLD-1 cells were plated in a 6-well plate, and a 10-μl pipette tip was used to generate a straight-line wound. The wells were washed with phosphate-buffered saline (PBS) and replenished with serum-free medium. Subsequently, the wounds were photographed at 0 h and 48 h after the injury. ImageJ software was used to measure the width of the gap.

### Immunofluorescence assay

Immunofluorescence assays were performed in 96-well plates (Catalog No. 6055300, PerkinElmer, Waltham, MA), with 1 × 10^4^ cells plated and incubated for 24 h. Subsequently, the cells were fixed with 4% PFA for 15 min, washed three times with PBS, and permeabilized and blocked with 0.5% Triton X-100 (Catalog No. T8200, Solarbio, Beijing, China) for 5 min. The cells were then incubated with Actin-Tracker Red-594 (Catalog No. C2205S, Beyotime) for about 30 min, followed by incubation with 4′,6-diamidino-2-phenylindole (DAPI; Catalog No. C0060, Solarbio) for about 5 min. Images were taken with an Opera Phenix Plus (Catalog No. HH14001000, PerkinElmer) and quantified with Harmony software.

### Coimmunoprecipitation

The cells were lysed in RIPA buffer on ice for 30 min and then centrifuged at 20,000 *g* for 10 min. Anti-Flag Magnetic Beads (Catalog No. HYK0207, MedChemExpress, Monmouth Junction, NJ) were utilized to pull down DBN1-Flag. The interacting proteins were eluted using 1× SDS loading buffer (Catalog No. P0015A, Beyotime) and subjected to heating at 95°C for 5 min. The products were subsequently analyzed either through Western blotting or LC-MS/MS.

## Ethical statement

All studies performed were approved by the Research Ethics Committee of Biology Research, West China Hospital at Sichuan University [Approval No. 2020 (374)]. Written informed consent and approval were obtained from all participants.

## Supplementary Material

qzae053_Supplementary_Data

## Data Availability

The MS proteomic data of CRC patients have been deposited in the ProteomeXchange Consortium via the iProX partner repository (ProteomeXchange: PXD039360). The MS phosphoproteomic data of CRC patients and CRC cells have been deposited in the ProteomeXchange Consortium via the iProX partner repository (ProteomeXchange: PXD042746). The raw sequence data reported in this study have been deposited in the Genome Sequence Archive for Human [83] at the National Genomics Data Center, Beijing Institute of Genomics, Chinese Academy of Sciences / China National Center for Bioinformation (GSA-Human: HRA003386), and are publicly accessible at https://ngdc.cncb.ac.cn/gsa-human.

## Code availability

The source code is freely available at https://ngdc.cncb.ac.cn/biocode/tools/BT007400.

## CRediT author statement

**Rou Zhang:** Investigation, Validation, Visualization, Writing – original draft. **Meng Hu:** Software, Formal analysis, Visualization, Writing – original draft. **Yu Liu:** Investigation, Validation, Visualization. **Wanmeng Li:** Visualization. **Zhiqiang Xu:** Investigation. **Siyu He:** Visualization. **Ying Lu:** Visualization. **Yanqiu Gong:** Validation. **Xiuxuan Wang:** Validation. **Shan Hai:** Resources, Funding acquisition. **Shuangqing Li:** Resources, Funding acquisition. **Shiqian Qi:** Supervision. **Yuan Li:** Resources. **Yang Shu:** Data curation, Software. **Dan Du:** Resources. **Huiyuan Zhang:** Resources. **Heng Xu:** Resources, Funding acquisition. **Zongguang Zhou:** Resources, Funding acquisition. **Peng Lei:** Resources, Project administration, Conceptualization. **Hai-Ning Chen:** Resources, Writing – review & editing, Supervision. **Lunzhi Dai:** Conceptualization, Writing – review & editing, Supervision, Project administration, Funding acquisition. All authors have read and approved the final manuscript.

## ORCID

0009-0001-5056-8345 (Rou Zhang)

0000-0001-5113-2888 (Meng Hu)

0000-0001-5359-1906 (Yu Liu)

0009-0009-1357-3629 (Wanmeng Li)

0000-0002-8133-1228 (Zhiqiang Xu)

0009-0007-4350-4232 (Siyu He)

0000-0002-6424-5424 (Ying Lu)

0000-0001-7890-5158 (Yanqiu Gong)

0000-0002-4925-7048 (Xiuxuan Wang)

0009-0007-7718-4249 (Shan Hai)

0000-0001-6889-9997 (Shuangqing Li)

0000-0002-7589-8877 (Shiqian Qi)

0000-0003-0274-855X (Yuan Li)

0000-0002-8304-1531 (Yang Shu)

0000-0001-9745-8197 (Dan Du)

0000-0002-1532-2462 (Huiyuan Zhang)

0000-0002-7748-2621 (Heng Xu)

0000-0002-7616-1199 (Zongguang Zhou)

0000-0001-5652-1962 (Peng Lei)

0000-0003-0104-8498 (Hai-Ning Chen)

0000-0002-3003-8910 (Lunzhi Dai)
