# Supplementary material for: Integrative Omics Uncovers Low Tumorous Magnesium Content as A Driver Factor of Colorectal Cancer
Source: Genomics Proteomics Bioinformatics. 2024 Jul 25;22(4):qzae053. doi: 10.1093/gpbjnl/qzae053 (PMC11514849; doi:10.1093/gpbjnl/qzae053)
Supplement: qzae053_Supplementary_Data [file qzae053_supplementary_data.zip › supplementary material captions.docx]

**Supplementary material**

**Figure S1 Quality control analysis of proteome and phosphoproteome data and Mg content detection**

**A.** Workflow of the proteome and phosphoproteome experiments. **B.** Pearson’s correlation analysis of 15 QC samples of the proteome to evaluate machine stability. A scatterplot matrix was generated for pairwise calculation of Pearson’s correlation coefficients among the samples, with density plots on the diagonal and correlation values displayed in the upper triangle. **C.** Pearson’s correlation analysis of 27 ISs to evaluate the robustness of TMT-based quantification in the proteomic study. Top right: pairwise calculation of Pearson’s correlation coefficients among the 27 ISs. Bottom left: elliptical chart showing pairwise comparisons of the 27 ISs. **D.** Pearson’s correlation analysis of replicate samples. **E.** Distribution of unique peptides among 27 TMT-labeled batches. **F.** Protein counts of each batch (light blue), counts of common proteins (blue), and cumulative protein counts among the 27 batches. **G.** Pearson’s correlation analysis of 6 QC samples of the phosphoproteome to evaluate machine stability. A scatterplot matrix was generated for pairwise calculation of Pearson’s correlation coefficients among the samples, with density plots on the diagonal and correlation values displayed in the upper triangle. **H.** Pearson’s correlation analysis of 20 ISs to evaluate the robustness of TMT-based quantification in the phosphoproteomic study. Top right: pairwise calculation of Pearson’s correlation coefficients among the 20 ISs. Bottom left: elliptical chart showing pairwise comparisons of the 20 ISs. **I.** Identified (blue) and quantified (orange) phosphoprotein counts of each batch and cumulative phosphoprotein counts among 20 batches. **J.** Number of identified and quantified phosphosites (blue) and phosphoproteins (red). **K.** Localization and distribution of phosphosites. **L.** Statistics of the number of phosphosites in a protein. **M.** Coefficient of variation of Mg content for the QC samples. QC, quality control; TMT, tandem mass tag; ISs, internal standards.

**Figure S2 Association analysis between Mg content and TNM stage (or OS)**

**A.–C.** Forest plots of univariate Cox models for clinical information for CRC patients in all samples (A), left-sided (B), or right-sided (C) colon samples. The data are presented as HR ± 95% confidence intervals. **D.**–**F.** Multivariate Cox regression models were designed to test for prognostic factors. Low Mg content was an independent predictor of outcome in all CRC patients (D) and right-sided CRC patients (E) but not in left-sided CRC patients (F). Data are presented as HR ± 95% confidence intervals. **, *P* < 0.01; *, *P* < 0.05; ns, no significance. Wilcoxon rank-sum test. **G.**–**I.** Mg content among all four TNM stages in tumors and DNTs of all samples (G), left-sided (H), or right-sided (I) colon samples. *, *P* < 0.05; ns, no significance. Wilcoxon rank-sum test. CI, confidence interval; HR, hazard ratio; ANOVA, analysis of variance; ALT, alanine transaminase; GGT, γ-glutamyltransferase; IDIL, indirect bilirubin; AST, aspartate transaminase; DBIL, direct bilirubin; TBIL, total bilirubin.

**Figure S3 Analysis of the Mg-associated proteome and phosphoproteome**

**A.** GOBP term enrichment analysis of proteins negatively associated with Mg content using the DAVID database. **B.** GOBP term enrichment analysis of proteins positively associated with Mg content using the DAVID database. **C.** The expression of complement-associated proteins in tumors and paired DNTs. **D.** The expression of cell adhesion-associated proteins in tumors and paired DNTs. DAVID, the Database for Annotation, Visualization and Integrated Discovery; GOBP, Gene Ontology biological process.

**Figure S4 Proteomic classification of CRC tumors**

**A.** Consensus matrix of unsupervised clustering based on the top 25 most variable proteins (k = 2, k = 3, and k = 4). **B.** The consensus CDF of unsupervised clustering based on the top 25 most variable proteins. **C.** Delta area (change in CDF area) plots for 2**–**10 clusters. **D.** Kaplan–Meier curves of OS for each proteomic subtype. **E.** Boxplot showing the differences in Mg content among proteomic subtypes. ****, *P* < 0.0001; ns, no significance. Wilcoxon rank-sum test. **F.** GOBP enrichment analysis of proteins upregulated in subtype II (subtype II *vs*. subtype I and subtype III) using DAVID. CDF, cumulative distribution function.

**Figure S5**  **Integrative analysis of proteome and phosphoproteome data**

**A.** Comparison between Mg-protein correlations and Mg-phosphosite correlations. *P* < 0.05. Spearman’s rank correlation test. **B.** and **C.** Top 20 phosphosites positively (B) or negatively (C) associated with Mg content independent of protein level. **D.** GOBP enrichment analysis of phosphoproteins with Mg negatively associated phosphosites. **E.** GOBP enrichment analysis of phosphoproteins with Mg positively associated phosphosites.

**Figure S6 Mg-regulated phosphosites related to cell adhesion**

**A.** Survival analysis of 79 CRC patients with different ARHGAP1^S51p^, FLNA^S1459p^, and TNS1^S1177p^ levels in tumors. Log-rank test. **B.** Effect of MgCl_2_ treatment on the formation of F-actin determined by immunofluorescence assays in DLD-1 cells. Scale bars, 500 μm. *, *P* < 0.05, Student’s *t* test. **C.** Immunoblotting assays were used to determine the expression of ARHGAP1 mutants. **D.** Transwell assays were performed for cells transfected with empty vector, ARHGAP1^WT^, ARHGAP1^S51A^, or ARHGAP1^S51D^. Scale bars, 500 μm. *, *P* < 0.05. Student’s *t* test. **E.** Effect of the empty vector, ARHGAP1^WT^, ARHGAP1^S51A^, and ARHGAP1^S51D^ on F-actin formation determined by immunofluorescence assays. Scale bars, 500 μm. *, ns, no significance. Student’s *t* test.

**Figure S7 DBN1-binding proteins affected by DBN1^S142p^ and Mg^2+^**

**A.** and **B.** Venn diagram showing the number of proteins strongly and specifically bound to DBN1^S142D^ (A) and DBN1^S142A^ (B), respectively. The fold changes in protein expression were > 1.2 between groups, *P* < 0.05. Student’s *t* test. Gene annotation enrichment analysis of the proteins obtained via protein**–**protein interaction network analysis. **C.** Schematic of the process of screening Mg-regulated DBN1-binding proteins affected by DBN1^S142p^. **D.** GO enrichment analysis of Mg-regulated DBN1-binding proteins. **E.** and **F.** Venn diagram showing the number of interacting proteins affected by both DBN1^S142p^ and Mg^2+^. **G.** The levels of the DBN1-binding protein CDC42 under different treatment and immunoprecipitation conditions. ****, *P* < 0.0001; **, *P* < 0.01; *, *P* < 0.05; ns, no significance. Student’s *t* test. **H.** Immunoblots showing the expression of ACTN4. MCODE, molecular complex detection.

**Figure S8 Mg-associated cell**–**matrix adhesion proteins as drug targets**

List of clinical drugs for the negatively Mg-associated cell–matrix adhesion proteins. Spearman’s rank correlation analysis, *P* < 0.05 indicating a significant correlation. The gender, TNM stage, survival event, age, and Mg content are annotated above the heatmap. The heatmap depicts the intensity of proteins using the z-score. The corresponding subcellular location (middle) and clinical drugs (right) used are listed. FDA, Food and Drug Administration.

**Table S1 Clinical information of CRC patients and Mg content in tumors and DNTs**

**Table S2 Genomic data of CRC patients**

**Table S3 Proteomic data of CRC patients**

**Table S4 Spearman’s rank correlation analysis of the levels of cell adhesion-related proteins and Mg content**

**Table S5 Phosphoproteomic data of CRC patients**

**Table S6 Phosphoproteomic data of Mg-treated HCT116 cells**

**Table S7 The proteins bound to DBN1 and affected by DBN1^S142p^ and Mg^2+^**

**Table S8 Subcellular location of proteins and their clinical drugs**
